# *In vivo* levels of mitochondrial hydrogen peroxide increase with age in mtDNA mutator mice

**DOI:** 10.1111/acel.12212

**Published:** 2014-03-13

**Authors:** Angela Logan, Irina G Shabalina, Tracy A Prime, Sebastian Rogatti, Anastasia V Kalinovich, Richard C Hartley, Ralph C Budd, Barbara Cannon, Michael P Murphy

**Affiliations:** 1 MRC Mitochondrial Biology UnitWellcome Trust/MRC Building, Cambridge, CB2 0XY, UK; 2 Department of Molecular Biosciences, the Wenner-Gren Institute, the Arrhenius Laboratories F3, Stockholm UniversityStockholm, SE-106 91, Sweden; 3 Centre for the Chemical Research of Ageing, WestCHEM School of Chemistry, University of GlasgowGlasgow, G12 8QQ, UK; 4 Vermont Center for Immunology & Infectious Diseases, The University of Vermont College of MedicineD-305 Given Building, Burlington, VT, 05405-0068, USA

**Keywords:** hydrogen peroxide, MitoB, mitochondria, mitochondrial DNA, mtDNA mutator mice

## Abstract

In mtDNA mutator mice, mtDNA mutations accumulate leading to a rapidly aging phenotype. However, there is little evidence of oxidative damage to tissues, and when analyzed *ex vivo,* no change in production of the reactive oxygen species (ROS) superoxide and hydrogen peroxide by mitochondria has been reported, undermining the mitochondrial oxidative damage theory of aging. Paradoxically, interventions that decrease mitochondrial ROS levels *in vivo* delay onset of aging. To reconcile these findings, we used the mitochondria-targeted mass spectrometry probe MitoB to measure hydrogen peroxide within mitochondria of living mice. Mitochondrial hydrogen peroxide was the same in young mutator and control mice, but as the mutator mice aged, hydrogen peroxide increased. This suggests that the prolonged presence of mtDNA mutations *in vivo* increases hydrogen peroxide that contributes to an accelerated aging phenotype, perhaps through the activation of pro-apoptotic and pro-inflammatory redox signaling pathways.

In mtDNA mutator mice (Trifunovic *et al*., 2004; Kujoth *et al*., 2005), the proof-reading domain of mitochondrial DNA polymerase γ is disrupted by a point mutation (D257A) and ‘knocked in’ to all tissues (Trifunovic *et al*., 2004; Kujoth *et al*., 2005). This leads to the accumulation of mtDNA mutations with age, giving rise to defective mitochondria and an accelerated aging phenotype that is evident from ~5 months (Trifunovic *et al*., 2004; Kujoth *et al*., 2005; Edgar *et al*., 2009; Hiona *et al*., 2010). The mitochondrial free radical theory of aging suggested that superoxide production by the respiratory chain leads to mtDNA damage, resulting in the assembly of defective respiratory chains that produce more superoxide, establishing a vicious cycle that underlies aging (Harman, 1972). However, most studies have reported negligible increases in oxidative damage in mutator mice (with one exception of a modest, ~19%, increase in protein carbonyls (Dai *et al*., 2010)), or expression of antioxidant enzymes, and the levels of ROS produced by mutator mitochondria or embryonic fibroblasts have been reported to be the same as controls when analyzed *ex vivo*, despite the accumulation of mtDNA mutations (Kujoth *et al*., 2005; Trifunovic *et al*., 2005; Hiona *et al*., 2010). These findings suggested that a vicious cycle of disruptive mitochondrial oxidative damage does not underlie normal aging (Loeb *et al*., 2005; Vermulst *et al*., 2007).

Recently, however, studies have shown that elevated mitochondrial ROS may yet contribute to aging in mutator mice. Expression of catalase within mitochondria (Dai *et al*., 2010), treatment with N-acetyl cysteine (Ahlqvist *et al*., 2012), or endurance exercise (Safdar *et al*., 2011) all delayed the onset of aging. All these interventions can decrease mitochondrial superoxide (Murphy, 2009; Cochemé *et al*., 2011), suggesting that mitochondrial ROS may contribute to the aging of mutator mice *in vivo*, but without changing *ex vivo* mitochondrial ROS or markedly increasing oxidative damage.

An insight that may reconcile these apparently contradictory findings is that mitochondrial ROS production *in vivo* is determined by respiratory chain redox state, substrate supply, ATP turnover, and membrane potential, which are all dependent on the ever-changing physiological conditions *in vivo*, compared to the stable environment in which isolated mitochondria are analyzed (Murphy, 2009). Furthermore, mitochondrial hydrogen peroxide is a redox signal that can alter cell function at levels too low to cause oxidative damage (Finkel, 2011).

To test whether the accumulation of mtDNA mutations in mutator mice increased ROS production, we measured hydrogen peroxide *in vivo* using the mitochondria-targeted mass spectrometry probe MitoB (Cochemé *et al*., 2011, 2012) (See Appendix S1). MitoB accumulates rapidly and extensively within mitochondria *in vivo* following IV injection (Porteous *et al*., 2013), where it reacts with hydrogen peroxide to form the diagnostic exomarker MitoP [although a contribution from peroxynitrite cannot be excluded (Cochemé *et al*., 2011, 2012)]. The mice are maintained for 6 h after MitoB injection, allowing a MitoP/MitoB ratio to develop that reflects the average mitochondrial hydrogen peroxide level *in vivo* under normal conditions. The tissue MitoP/MitoB ratio is then determined by liquid chromatography followed by tandem mass spectrometry (Cochemé *et al*., 2011, 2012; Chouchani *et al*., 2013).

There was no difference in MitoP/MitoB ratio between young mutator mice and age-matched controls (Fig. 1A), even though mtDNA mutations are ~5-fold greater in mutator mice at this age. Therefore, mtDNA mutation in itself is not sufficient to increase mitochondrial ROS *in vivo*. For mature (35–42 weeks) mutator mice, where accelerated aging is evident, the MitoP/MitoB ratio increased in the heart and kidney compared to age-matched controls (Fig. 1B). There was also an increase in MitoP/MitoB ratio for heart, kidney, liver, and skeletal muscle when mature and young mutator mice were compared (Fig. 1C). (For the liver, there was an unexplained bimodal distribution, but the difference remained statistically significant (*P* < 0.05) when the upper four points were excluded.) In contrast, the MitoP/MitoB ratio did not increase for the same age change in control mice (Fig. 1D, young vs. mature). As mature mutator mice are close to the end of their lives, we also assessed the MitoP/MitoB ratio in old control mice (111 weeks) at a similar life stage to mature mutator mice. The MitoP/MitoB ratio did not increase with age, comparing mature and old control mice (Fig. 1D). Therefore, the MitoP/MitoB ratio increases in mutator mice with age to a greater extent than in control mice, when compared by either chronological age or life stage.

**Figure 1:**
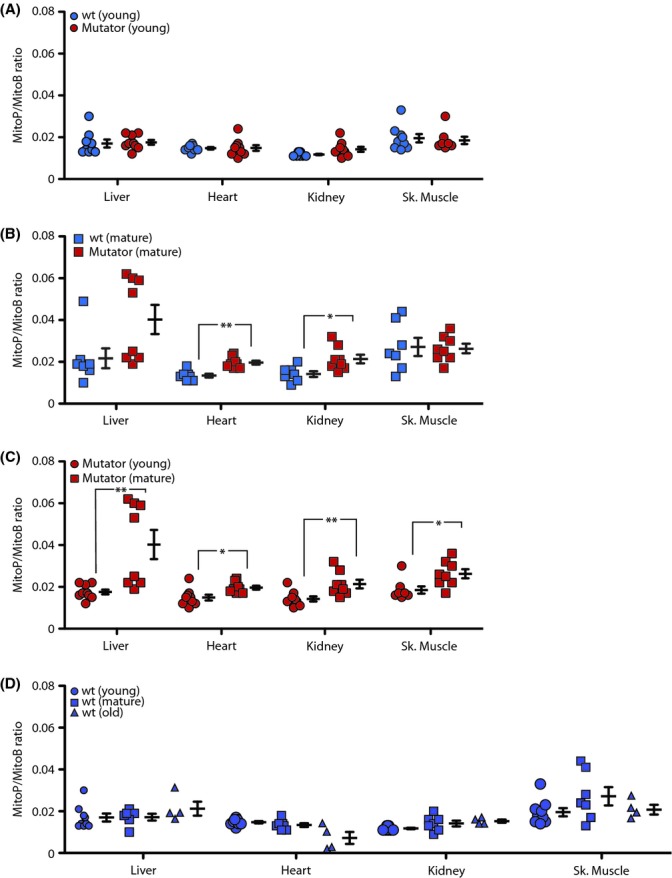
Hydrogen peroxide levels in mutator and control mice. Mice were injected with MitoB, and 6 h later, MitoB and MitoP levels were analyzed. Each point represents one mouse; data are means ± SEM of 7–9 mice. Statistical significance was determined using Student’s *t*-test: *P < 0.05, **P < 0.01. (A) young (6–20 weeks), (B) mature (35–42 weeks), (C) young and mature mutator mice, (D) young, mature or old (111 weeks) control mice.

Mitochondrial tissue content was similar for all the mice (Fig. S1A, B). The MitoB uptake into tissues was not different between the young mutator and control mice (Fig. S1C), but was lower for mature mutator mice compared to mature control mice (Fig. S1D), perhaps reflecting a decrease in mitochondrial membrane potential in mutator mice with age. However, as MitoP formation is normalized to MitoB (Cochemé *et al*., 2011), this does not affect the MitoP/MitoB ratio, as was confirmed for the experiments reported here (Fig. S1E). Therefore, these changes in MitoP/MitoB ratio reflect an increase in mitochondrial hydrogen peroxide within mutator mice as they age.

The increase in mitochondrial hydrogen peroxide with age in the mutator mice *in vivo* may arise from disruption to mitochondrial function due to mtDNA mutations increasing superoxide production, which is suppressed *in vitro* by saturation with substrates and oxygen. As oxidative damage does not accumulate markedly in mutator mice, the mtDNA mutations may activate mitochondrial ROS production as part of subtle redox signaling pathways that contribute to accelerated aging. Increased apoptosis is associated with elevated mitochondrial ROS, consistent with greater cell death in mutator mice (Kujoth *et al*., 2005; Hiona *et al*., 2010; Ahlqvist *et al*., 2012). A further possibility is that mtDNA damage modifies the response of the immune system, perhaps by triggering the NLRP3 inflammasome, which requires elevated mitochondrial ROS (Green *et al*., 2011). To assess this possibility, we measured the response to immune stimulation with lipopolysaccharide (LPS) of young and mature mutator and control mice (Fig. 2). The cytokine profiles of young control and mutator mice were similar following LPS stimulation (Fig. 2). However, for mature mice, the increase in some pro-inflammatory cytokines following LPS stimulation was markedly higher in the mutator compared to control mice (Fig. 2), notably for IFN-γ, TNF-α, IL-1β, and IL-10. Similar enhanced inflammatory activity in response to stimulation is seen with age in control animals (Tateda *et al*., 1996; Bruunsgaard *et al*., 2001). Thus, one possibility is that the activation of mitochondrial redox signaling in response to exposure to chronic mtDNA damage generates a pro-inflammatory environment that contributes to the aging phenotype in mutator mice.

**Figure 2:**
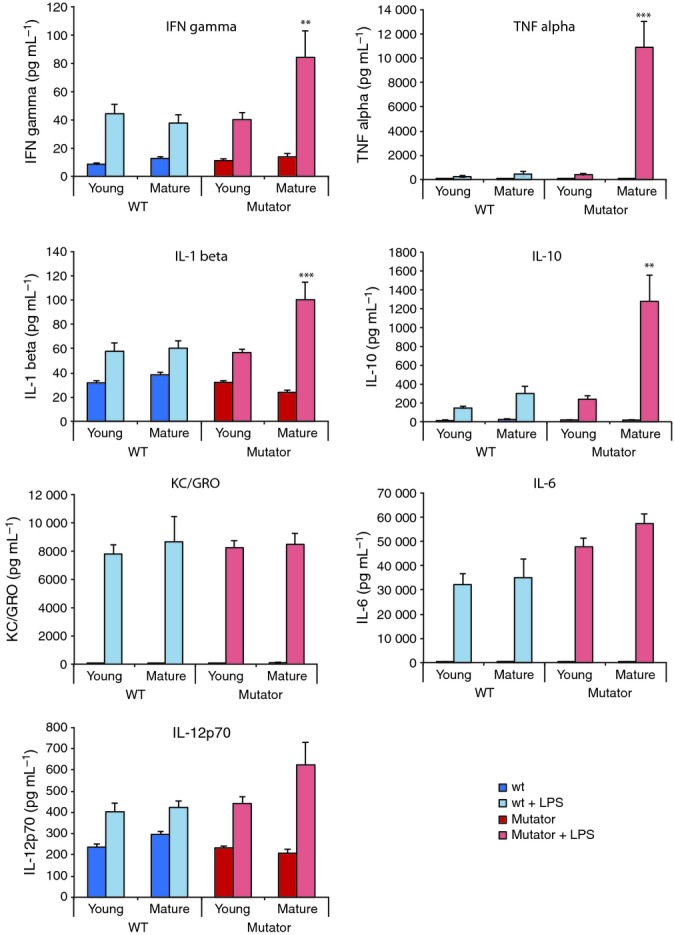
Measurement of serum cytokines in young (10–16 weeks) and mature (40–48 weeks) mutator and age-matched control mice following injection with LPS or with saline carrier. Data are means ± SEM of 5–7 mice per treatment group. Statistical significance was determined between the LPS-treated young and mature groups for the mutator and control mice using Student’s *t*-test: **P < 0.01, ***P < 0.001. IL, interleukin; IFN, interferon; TNF, tumor necrosis factor; KC/GRO, keratinocyte-derived cytokine.

Our findings show that the presence of mtDNA mutations in mutator mice increases mitochondrial ROS levels with age *in vivo*, and suggest that the ROS production contributes to the accelerated aging phenotype. Accelerated aging may occur through the activation of pro-apoptotic and pro-inflammatory redox signaling pathways in response to mtDNA damage. Whether these mechanisms drive aging in general, are specific to the mtDNA mutator mouse model, or are merely secondary consequences of aging are important questions to be addressed in future work.

## Supporting Information

**Fig. S1** Measurement of citrate synthase and MitoB levels in mouse tissues. **Appendix S1** Experimental Procedures.
