# Supplementary material for: In vivo levels of mitochondrial hydrogen peroxide increase with age in mtDNA mutator mice
Source: Aging Cell. 2014 Mar 13;13(4):765–8. doi: 10.1111/acel.12212 (PMC4326952; doi:10.1111/acel.12212)
Supplement: Supplementary file 1 — Fig. S1 Measurement of citrate synthase and MitoB levels in mouse tissues. Appendix S1 Experimental Procedures. [file acel0013-0765-sd1.pdf]

Supporting Information

Appendix S1

Experimental Procedures

Mouse experiments

The mtDNA mutator mice were derived from the Larsson laboratory (Trifunovic *et al.* 2004), backcrossed in Stockholm onto the C57Bl/6 background for 10 generations and maintained as heterozygous crosses with wild-type females from another colony, in order to avoid any impact of maternally transmitted mtDNA mutations. The mice used in this study homozygously and ubiquitously expressed the knocked-in mutated version of mitochondrial DNA polymerase γ containing a single point mutation (D257A) in the proof-reading 3'-5' exonuclease domain. Control mice were littermates homozygous for the wild type allele. Mice were maintained at 21 ˚C with free access to water and standard lab chow, and powdered food was added for all mice at age 25 weeks or older. When assessed with MitoB young mutator mice were 6 – 19 weeks of age and mature mutator mice were 35 – 42 weeks of age. The age-matched control mice were used at 6-20 weeks of age for young, 34-45 weeks for mature and 111 weeks for old. Data from both sexes are pooled for each group; sexes were not assessed separately due to insufficient numbers for comparison.

We measured mitochondrial hydrogen peroxide *in vivo* using the MitoB mass spectrometric probe as described previously (Cocheme *et al.* 2011; Cocheme *et al.* 2012; Chouchani *et al.* 2013). Briefly, 75 nmol MitoB (~ 3 μmol kg^{-1} for 25 – 30 g mice) in 50 μl saline was administered by tail vein injection to the mouse by restraining the mouse. Injections took place between 10 am and 12 noon and the mice were returned to their cages and returned to normal levels of activity for 6 h. At the end of this time the mice were killed by cervical dislocation and tissues (heart, liver, kidney and skeletal muscle (gastrocnemius)) were harvested and flash frozen on liquid nitrogen. To do this the tissue was dissected and within 1 sec was flash frozen between two aluminum blocks held at -190 ºC with liquid N_2. This thin layer of tissue was then kept immersed in liquid nitrogen in a small mortar until all tissues were dissected and similarly flash frozen. Then all tissues were wrapped in pre-labelled aluminum foil and placed in liquid nitrogen. Tissues were then stored at –80°C and transported to Cambridge, UK on dry ice for analysis. Blood was taken from the hearts of mice just immediately after euthanasia, proteinase inhibitors were added and serum was frozen and stored at -80˚C. For the analysis of cytokines, the mice were injected intraperitoneally with 20 μg of LPS (*Escherichia coli* 055:B5 from Sigma L6529-1MG) in 100 μl sterile PBS and compared with mice that were injected with saline alone. 187 ± 35 minutes (mean ± sd, n = 25) after injection the mice were euthanized and plasma samples isolated and analysed for cytokine content. All experiments were approved by the animal ethics committee of the North Stockholm region (N31/12).

Analysis of tissue levels of MitoB and MitoP

MitoB and MitoP were extracted from tissues and assessed by LC-MS/MS as previously described (Cocheme *et al.* 2011; Cocheme *et al.* 2012; Chouchani *et al.* 2013). Briefly, ~50 mg tissue were homogenised, spiked with deuterated internal standards, and MitoB and MitoP were extracted using acetonitrile/formic acid. MitoP and MitoB were detected using an I-class Acquity LC attached to a Xevo TQ-S triple quad mass spectrometer (Waters), analysed using MassLynx software, and the amounts of MitoP and MitoB in each sample was determined relative to a standard curve. The MitoP/MitoB ratios for each sample were then calculated. One of the 8 mature control mice showed anomalously high levels of MitoB in the liver, kidney and heart (3.5, 1.8 and 2.6-fold greater than the means of the other 7 mice in this cohort, with the increases over the means 5.9x, 3.4x and 6.9x of the standard deviation of the rest of the cohort). The MitoB injection for this mouse was therefore judged to be unreliable and data from this mouse were excluded from the analysis.

Analysis of citrate synthase

Tissues, that had been stored frozen until required, were homogenised in 50 mM Tris-HCl, 2 mM sodium citrate, 0.6 mM $MnCl_2$, pH 7 and stored frozen until required. Citrate synthase was assessed as described (Robinson Jr. *et al.* 1987). Protein concentration was determined by the BCA assay.

Analysis of serum cytokines

Plasma cytokine levels were measured by the Core Biochemical Analysis Laboratory at Addenbrooke's Hospital, Cambridge, using a multiplexed electrochemical luminescence immunoassay on an automated biochemical analyser (MesoScale Discovery Sector 6000 analyser, Gaithersburg, MD, USA). Samples were analysed using a 7-plex proinflammatory cytokine high sensitivity kit (product code K15012C-2, MesoScale Discovery) which evaluated interleukin IL-1β, IL-6, IL-10, IL-12, tumour necrosis factor alpha (TNF-α), interferon gamma (IFNγ) and chemokine KC/GRO. All reagents and calibration standards were supplied by the manufacturers. A stock solution of recombinant cytokines was used to produce a standard curve against which sample concentrations were determined.

Supplementary References

Chouchani ET, Methner C, Nadtochiy SM, Logan A, Pell VR, Ding S, James AM, Cocheme HM, Reinhold J, Lilley KS, Partridge L, Fearnley IM, Robinson AJ, Hartley RC, Smith RA, Krieg T, Brookes PS , Murphy MP (2013). Cardioprotection by S-nitrosation of a cysteine switch on mitochondrial complex I. *Nat Med*. **19**, 753-759.

Cochemé HM, Logan A, Prime TA, Abakumova I, Quin C, McQuaker SJ, Patel JV, Fearnley IM, James AM, Porteous CM, Smith RA, Hartley RC, Partridge L , Murphy MP (2012). Using the mitochondria-targeted ratiometric mass spectrometry probe MitoB to measure H2O2 in living Drosophila. *Nature protocols*. **7**, 946-958.

Cochemé HM, Quin C, McQuaker SJ, Cabreiro F, Logan A, Prime TA, Abakumova I, Patel JV, Fearnley IM, James AM, Porteous CM, Smith RA, Saeed S, Carre JE, Singer M, Gems D, Hartley RC, Partridge L , Murphy MP (2011). Measurement of H2O2 within living Drosophila during aging using a ratiometric mass spectrometry probe targeted to the mitochondrial matrix. *Cell metabolism*. **13**, 340-350.

Robinson Jr. JB, Brent LG, Sumegi B , Srere PA (1987). An enzymatic approach to the study of the Krebs tricarboxylic acid cycle. In *Mitochondria - a practical approach*. (VM Darley-Usmar, D Rickwood , MT Wilson, eds). Oxford: IRL Press, pp. 153-170.

Trifunovic A, Wredenberg A, Falkenberg M, Spelbrink JN, Rovio AT, Bruder CE, Bohlooly YM, Gidlof S, Oldfors A, Wibom R, Tornell J, Jacobs HT , Larsson NG (2004). Premature ageing in mice expressing defective mitochondrial DNA polymerase. *Nature*. **429**, 417-423.

Supplementary Figure 1

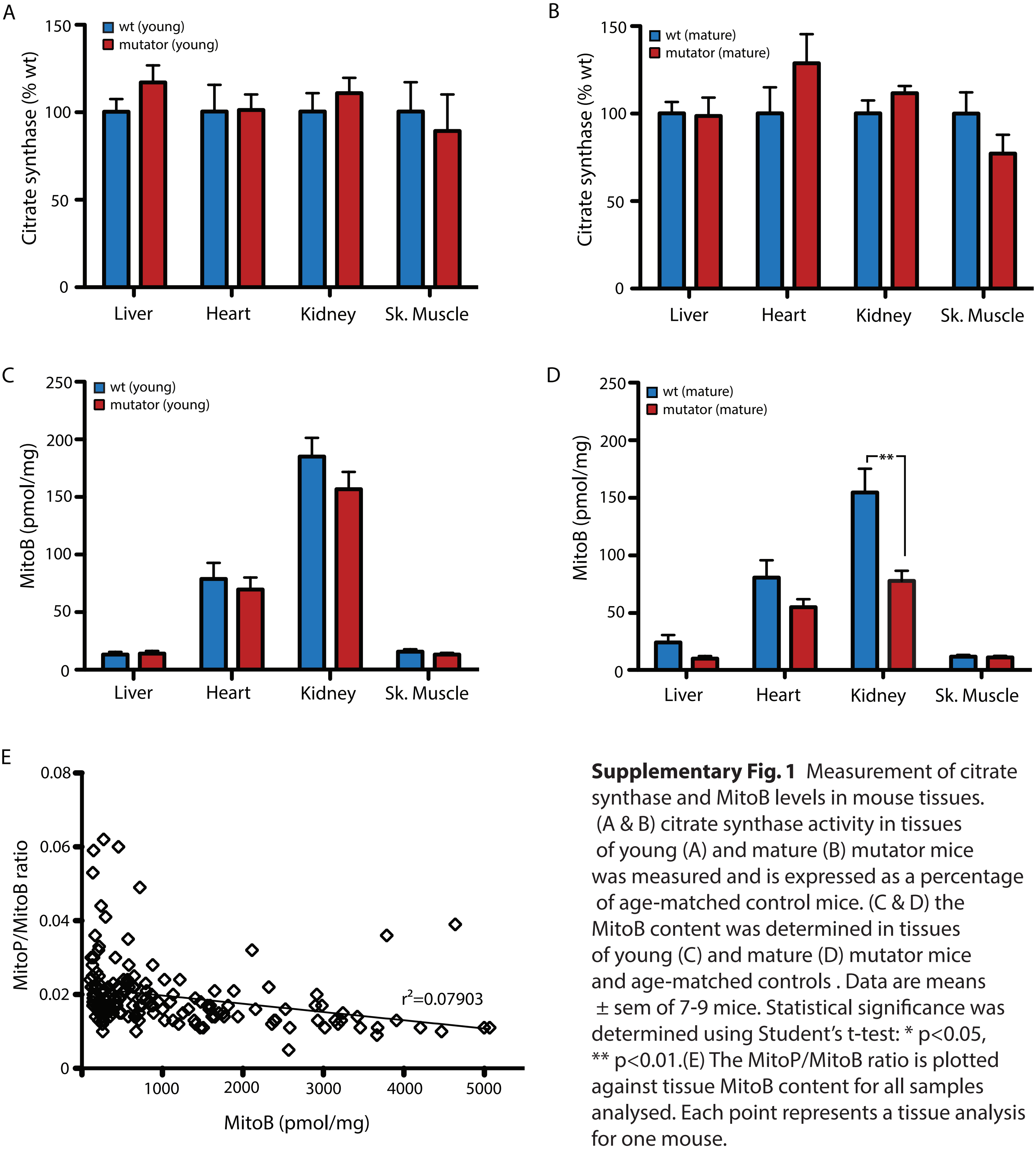

Supplementary Fig. 1 Measurement of citrate synthase and MitoB levels in mouse tissues. (A & B) citrate synthase activity in tissues of young (A) and mature (B) mutator mice was measured and is expressed as a percentage of age-matched control mice. (C & D) the MitoB content was determined in tissues of young (C) and mature (D) mutator mice and age-matched controls . Data are means ± sem of 7-9 mice. Statistical significance was determined using Student's t-test: * $p<0.05$, ** $p<0.01$.(E) The MitoP/MitoB ratio is plotted against tissue MitoB content for all samples analysed. Each point represents a tissue analysis for one mouse.
